# Bacterial Microbiome Dynamics in Post Pull-Through Hirschsprung-Associated Enterocolitis (HAEC): An Experimental Study Employing the Endothelin Receptor B-Null Mouse Model

**DOI:** 10.3389/fsurg.2018.00030

**Published:** 2018-04-06

**Authors:** Zhi Cheng, Lifu Zhao, Deepti Dhall, Paul M. Ruegger, James Borneman, Philip K. Frykman

**Affiliations:** ^1^Division of Pediatric Surgery and Department of Surgery, Cedars-Sinai Medical Center, Los Angeles, CA, United States; ^2^Department of Pathology, Cedars-Sinai Medical Center, Los Angeles, CA, United States; ^3^Department of Microbiology and Plant Pathology, University of California, Riverside, Riverside, CA, United States

**Keywords:** hirschsprung-associated enterocolitis, Hirschsprung enterocolitis, HAEC, pull-through surgery, microbiome, *Akkermansia*, bacteroidetes

## Abstract

**Purpose:**

Hirschsprung-associated enterocolitis (HAEC) is the most frequent potentially life-threatening complication in children with Hirschsprung disease (HSCR) even after definitive corrective surgery. Mounting evidence suggests that intestinal microbiota likely contribute to the etiology of enterocolitis, so the aim of this study was to use a mouse model of post pull-through HAEC to compare the fecal bacterial communities of animals which developed HAEC to those free of enterocolitis.

**Methods:**

Ten *Ednrb^*−*/*−*^* and 8wild type mice underwent the microsurgical pull-through surgery, and stool was collected at the time of surgery, and then either at 2 and 4 weeks after the operation, or when the mice developed enterocolitis. The mid-colon of all animals was collected, prepared and histologically graded for enterocolitis. Fecal DNA was isolated and bacterial 16S rRNA genes analyzed using Illumina sequencing.

**Results:**

Six *Ednrb^−/−^* mice developed HAEC with a mean enterocolitis score of 5.7, while the remaining 4 mutant and 8 WT mice remained free of enterocolitis by 4 weeks. The HAEC group had lower alpha diversity by Chao1 analysis compared with WT group, while the *Ednrb^−/−^* mice demonstrated distinct bacterial communities from WT mice on beta diversity analysis. The most striking finding was increased proportion of *Akkermansia* and reduced Bacteroidetes compared with the NO HAEC and WT groups, suggesting *Akkermansia* may contribute to development of enterocolitis while Bacteroidetes may be protective. Less abundant genera that were reduced in HAEC were *Dysgonomas* and *Clostridium* XIVa which may play a protective role.

**Conclusions:**

This is the first study to identify *Akkermansia* as potentially playing a role in HAEC, either as a pathobiont taxa contributing to pathogenesis of enterocolitis, or possibly a protective commensal taxa expanded in response to inflammation. These findings characterized the dynamic shifts in the gut microbial communities through the onset of post pull-through HAEC, and suggests that there may be identifiable bacterial community differences in HSCR patients that are high risk for developing HAEC.

## Introduction

Hirschsprung-associated enterocolitis (HAEC) is the most frequent potentially life-threatening complication of Hirschsprung disease (HSCR) patients (1). Reported incidence varies widely, ranging from 17–50% with most contemporary series reporting approximately 30–40% incidence in HSCR patients, with more than 80% occurring after pull-through surgery (2, 3). While the etiology of HAEC is not clear, recent clinical studies have identified marked fecal bacterial and fungal community differences in patients with HAEC compared with those free from enterocolitis (4–6). Some of the challenges with interpreting these human studies is variability between individual patient diets, antibiotic exposure, type of operation for HSCR, as well as lack of clear definition of what constitutes HAEC. To address some of these shortcomings, we created an experimental system to investigate HAEC bacterial community dynamics from the time of pull-through surgery to 4 weeks after surgery. Our lab employed a robust surgical model of post pull-through HAEC using Endothelin receptor B-null (*Ednrb^*−*/*−*^*) mouse model of Hirschsprung disease with partial colon aganglionosis, that underwent a single-stage microsurgical pull-through operation closely mimicking treatment of HSCR in infants (7, 8). Herein, we studied *Ednrb^*−*/*− *^*and wild type littermate control mice at the time of colon pull-through surgery, 2 and 4 weeks after surgery. We compared the colonic bacterial populations of mice which developed enterocolitis with those free of enterocolitis.

## Material and Methods

### Study Design

**Figure 1:**
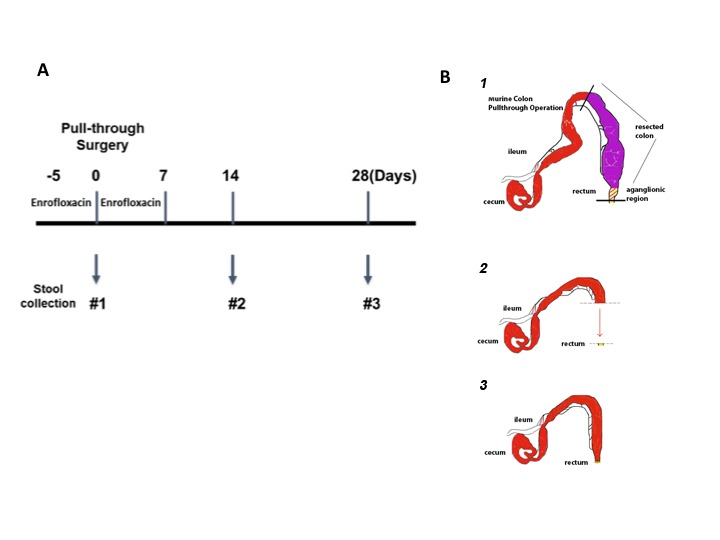
Experimental design and microsurgical pull-through operation. **(A)** The timeline for antibiotic administration, pull-through surgery and stool sample collection. Animals were given enrofloxacin drinking water for 5 days before pull-through surgery and continued for 7 days after surgery. The stool samples were collected at the time of surgery (#1), 14 days (#2), and 28 days (#3) post-surgery for those animals. **(B)** The steps of the microsurgical pull-through operation, demonstrating the (1) resection of the aganglionic region, (2) advancement of the normal colon and (3) anastomosis to the distal rectum.

The goal of this study was to explore the hypothesis that identifiable microbial factors may play a role in development of HAEC in our established murine model of post pull-through HAEC. A prospective observational design was employed with the pre-determined end points of either: (a) development of post pull-through HAEC within 28 days after surgery, or (b) event free survival of 28 days after surgery (Figure 1A). When post-surgical animals developed clinical signs of enterocolitis, they were euthanized and had stool and colon collected. Alternatively, post surgical animals that did not develop enterocolitis were sacrificed at 28 days, at which point stool and colon were collected for analysis.

### Animals

The research protocol and all animal care procedures were approved by the Institutional Animal Care and Use Committee (IACUC protocol #2302) at Cedars-Sinai Medical Center. Mice (*Ednrb^tm1Ywa^/J* on a hybrid C57BL/6J-129Sv background; JAX stock no. 003295 were purchased from Jackson Laboratory (Bar Harbor, ME) and housed at the animal facility of Cedars-Sinai Medical Center. The breeding scheme, animal care and genotyping were performed as previously described (7). *Ednrb^*−*/*−*^* mice with partial colonic aganglionosis and *Ednrb^+/+^* littermate control animals 21–42 days of age were used.

### Microsurgical Colon Resection and Pull-Through Operation, Clinical Assessment, Stool and Tissue Collection

Both genotypes of *Ednrb* mice were employed in this study: 8 *Ednrb^+/+^* mice (wild type, WT) and 10 *Ednrb^−/−^* mice and underwent the pull-through operation between 3 to 6 weeks of age. In preparation for the colon resection and pull-through operation, antibiotic drinking water containing enrofloxacin (0.25 mg/ml) was administered 5 days prior to surgery and then continued 7 days after the operation (Figure 1A), closely mirroring clinical practice in children with Hirschsprung disease. The microsurgical partial colon resection and pull-through operation, pre- and postoperative care has been described previously by our lab [Zhao et al. (7)] and a brief schematic of this procedure is shown in Figure 1B. Animals that survived through the perioperative period of 5 days were then followed prospectively for development of clinical signs of enterocolitis for 28 days. All study animals had health assessments daily including body weight monitored every 2 to 3 days. Any animal appearing clinically ill was euthanized when it developed clinical enterocolitis defined as: reduced activity and weight loss, and either new onset of loose stools or lack of stooling. Stool specimens were collected for each animal at two or three time points: the first was collected on all animals at time 0, (the time of pull-through surgery); the second either at 14 days after surgery, or at the time of enterocolitis diagnosis and euthanasia; and the third 28 days after surgery for those animals surviving to this end point (Figure 1A). All feces specimens were snap frozen in liquid nitrogen and stored at −80°C. The entire colon was collected and preserved in 10% neutral buffered formalin at room temperature for histopathological scoring for enterocolitis.

### Study Group Designations

WT = Wild type mice at time 0 (WT 0), 2 weeks (WT 2) and 4 weeks (WT 4) after pull-though surgery, none of which developed HAEC.

HAEC 0 = *Ednrb^−/−^* mice that went on to develop HAEC, but were free of HAEC at time 0.

HAEC 2 = *Ednrb^−/−^* mice which developed HAEC approximately 2 weeks after pull-through surgery;

NO HAEC 0 = *Ednrb^−/−^* mice which did not develop HAEC, and were free of HAEC at time 0.

NO HAEC 2 and 4 = *Ednrb^−/−^* mice which did not develop HAEC by 2 weeks, or 4 weeks after pull-through surgery, respectively.

### Histologic Preparation of Colon and Histopathological Scoring for Enterocolitis

**Figure 2:**
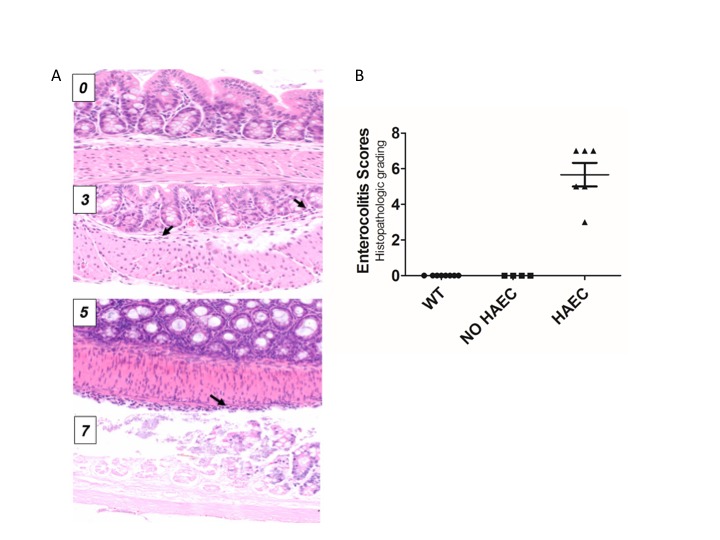
Histological scores elevated in Ednrb*^−^*^/^*^−^* mice with clinical enterocolitis. **(A)**, examples of histologic enterocolitis scores (H&E stained at 200×) ranging from 0 to 7. Total score 0 (normal); total score 3 (mild inflammatory infiltrate in the submucosa, inflammation 1, depth 2) arrows show neutrophils; total score 5 (mild inflammatory infiltrate at the serosal surface only, inflammation 1, depth 4) arrow show inflammatory cells at the serosal surface; (D) total score 7 (transmural necrosis, depth 4, inflammation 3). **(B)**, both wild type (WT) and *Ednrb^*−*/*−*^* NO HAEC groups had total enterocolitis score = 0, consistent with no evidence of clinical enterocolitis. The *Ednrb^*−*/*−*^* HAEC mice had increased total score 5.67 ± 0.67, indicating severe enterocolitis. *N* = 8, 4 and 6 for WT, NO HAEC and HAEC, respectively. One-way ANOVA analysis *P* < 0.0001.

Tissue from the mid-colon of all animals was paraffin embedded, sectioned, H&E stained and histopathologically graded for enterocolitis as previously described (9). The validated murine enterocolitis grading system assesses two components: severity of inflammation (inflammatory infiltrates and presence/absence of necrosis (0–3); and depth of inflammation (none, mucosa, submucosa, muscularis propria, subserosa, and serosa, 0–4, respectively) which are added together to determine the enterocolitis score (0–7) (Figure 2A).

### Stool DNA Isolation

Stool DNA isolation was performed using the PowerSoil DNA Isolation Kit (MO BIO Laboratories, Carlsbad, CA, USA), and a 30 s beat-beating step using a Mini-Beadbeater-16 (BioSpec Products, Bartlesville, OK, USA).

#### Illumina 16S rRNA Gene Sequencing

Illumina bacterial 16S rRNA gene libraries were constructed as follows. PCRs were performed in an MJ Research PTC-200 thermal cycler (Bio-Rad Inc., Hercules, CA, USA) as 100 µl reactions containing: 50 mM Tris (pH 8.3), 500 µg/ml bovine serum albumin (BSA), 2.5 mM MgCl_2_, 250 µM of each deoxynucleotide triphosphate (dNTP), 400 nM of the forward PCR primer, 200 nM of each reverse PCR primer, 2.5 µl of DNA template, and 0.625 units JumpStart Taq DNA polymerase (Sigma-Aldrich, St. Louis, MO, USA). PCR primers 515F (GTGCCAGCMGCCGCGGTAA) and 806R (GGACTACHVGGGTWTCTAAT) were used to targeted the 16S rRNA gene containing portions of the hypervariable regions V4 and V5, with the reverse primers including a 12 bp barcode (10). Thermal cycling parameters were 94°C for 5 min; 35 cycles of 94°C for 20 s, 50°C for 20 s, and 72°C for 30 s, and followed by 72°C for 5 min. PCR products were purified using the MinElute 96 UF PCR Purification Kit (Qiagen, Valencia, CA, USA).

Bacterial DNA sequencing (single-end 100 base) was performed using an Illumina HiSeq 2500 (Illumina, Inc., San Diego, CA). We used the USEARCH program (11) to perform de-multiplexing, quality filtering, OTU picking and assigning taxonomy using default parameters or recommended guidelines. Briefly, after demultiplexing and using the recommended 1.0 expected error threshold, sequences were filtered to keep 93.7% of reads. Sequences were then dereplicated and clustered into zero-radius OTUs using the UNOISE3 algorithm (12), which also detects and removes chimeric sequences. An OTU table was then generated using the otutab command. OTUs having non-bacterial DNA were identified by performing a local BLAST search (13) of their seed sequences against the nt database. OTUs were removed if any of their highest scoring BLAST hits contained taxonomic IDs within the family Rodents or to PhiX. Taxonomic assignments to the OTUs were performed with SINTAX (14) using RDP Classifier 16S training set number 16 (15) as the reference database.

#### Microbiome Data Analyses

For statistical and graphical representation of the data, the OTU table was rarified to an even depth of 200,000 reads per sample. Alpha diversity was assessed using QIIME 1.9.1 (16) and the default options of four metrics: Chao1, Shannon Index, Simpson Index and Faith’s Phylogenetic Diversity. Beta diversity was assessed using QIIME 1.9.1 to calculate a Hellinger beta diversity distance matrix, which was depicted using principle coordinates analysis (PCoA), and statistically assessed by performing Adonis tests. Taxonomic plots and tables were created using QIIME 1.9.1. Statistical differences for the alpha diversity and the taxonomic data were assessed by one-way analyses of variance (ANOVA) and Fisher's least significant difference (LSD) tests using Prism (GraphPad, La Jolla, CA). The bacterial sequences have been deposited in the National Center for Biotechnology Information (NCBI)’s Sequence Read Archive (SRA) under SRA Identifier Number SRP127505.

## Results

### Animals and Histological Scoring for HAEC

Ten *Ednrb^−/−^* and 8 WT mice underwent successful pull-through operation. Six *Ednrb^−/−^* mice developed HAEC 12–16 days after surgery with a mean enterocolitis score of 5.67 ± 0.67, while the remaining 4 mutant mice did not develop enterocolitis by 28 days (Figure 2B). None of the 8 WT mice developed HAEC.

## Bacterial Community Analyses

### Alpha Diversity

**Figure 3:**
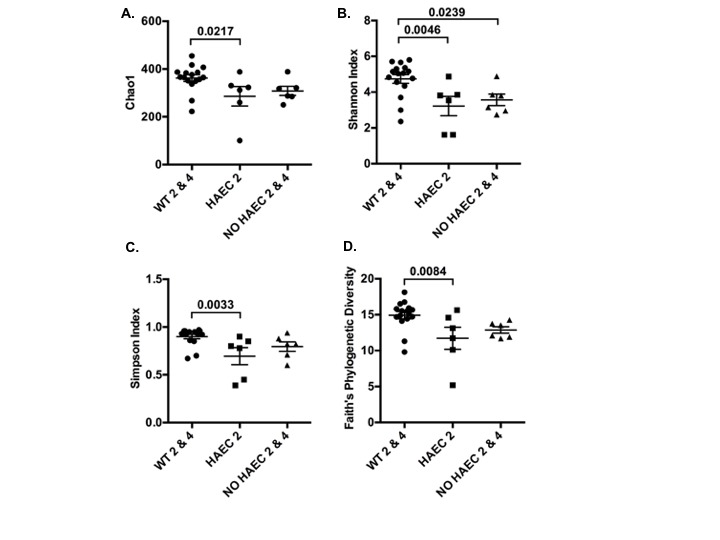
Alpha diversity of fecal bacteria measured at several times in relation to pull-through surgery from WT and *Ednrb*^*−/−*^ mice with and without HAEC. Four different metrics were used: **(A)** Chao1, **(B)** Shannon Index, **(C)** Simpson Index and **(D)** Faith’s Phylogenetic Diversity. P-values from ANOVAs are indicated above brackets. Bars are SE. *n* = 8 (WT 0, 2, 4), 6 (HAEC 0, 2), 4 (NO HAEC 0, 2), 2 (NO HAEC 4).

To examine alpha diversity between genotypes and phenotypes over time, we performed four alpha diversity analyses for each group. The estimated number of OTUs was lower in mice exhibiting enterocolitis (HAEC 2) than WT mice (WT 2 and WT 4) after surgery (Figure 3A). Similar relationships were obtain for three different metrics of alpha diversity (Figure 3B–D). Rarefaction curves (Figure S1) and analysis of the observed vs. estimate number of OTUs showed that on average 86.1% (data not shown) of the OTUs were identified by this analysis, demonstrating that this study identified most of the different types of bacteria in the samples.

### Beta Diversity

**Figure 4:**
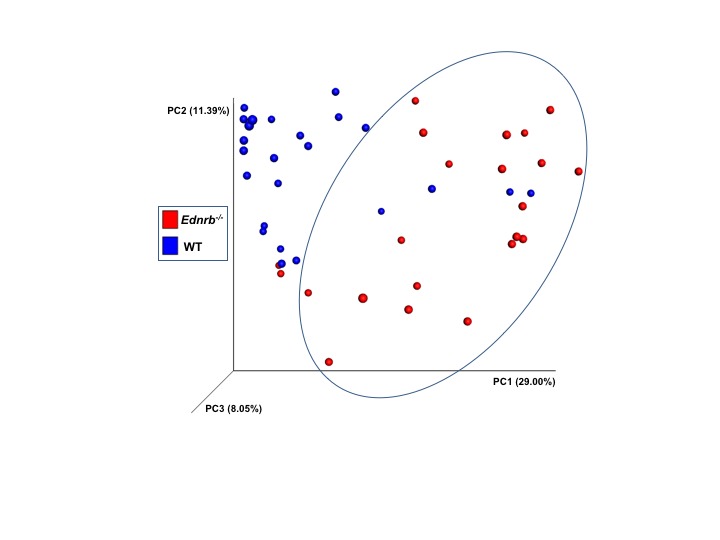
Bacterial community analyses of fecal bacteria at several times in relation to pull-through surgery from WT and *Ednrb*^−/−^ mice with and without HAEC. The figure is a principal coordinates analysis (PCoA) of Hellinger beta diversity values generated from 16S rRNA gene sequences (*n* = 22 and 24 for *Ednrb^−/−^* and WT mice, respectively).

We performed a community analysis of fecal bacteria from *Ednrb^−/−^* and WT mice from all 3 time points by using a principal coordinates analysis (PCoA) of Hellinger beta diversity values (Figure 4) and Adonis tests (Table S1). We found differences in the bacterial communities in the WT animals compared with the *Ednrb^−/−^* mice (*p* = 0.001) suggesting genotype is selecting for different fecal bacterial communities. Further beta diversity analyses and Adonis tests (Table S1) also show differences in the bacteria communities between WT 0, WT 2 and WT 4, suggesting the surgery itself changes fecal bacterial communities.

### Taxonomic Analysis

#### Phyla Taxa

**Figure 5:**
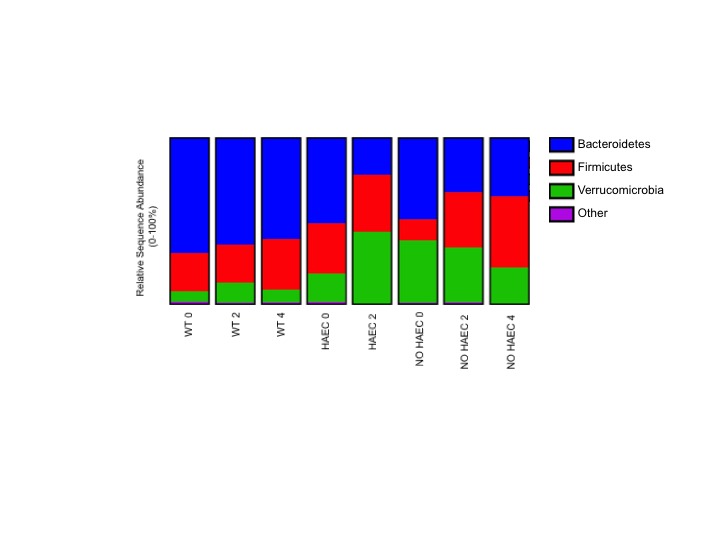
Fecal bacterial phyla at several times in relation to pull-through surgery from WT and *Ednrb*^*−/−*^ mice with and without HAEC. Data were generated by analyses of 16S rRNA gene sequences. *n* = 8 (WT 0, 2, 4), 6 (HAEC 0, 2), 4 (NO HAEC 0, 2), 2 (NO HAEC 4). See Supplemental Tables S2, S3 for more details including statistical analyses.

Next, we performed taxonomic analyses of each phenotype for each time point. We found a significant increase in proportion of phyla Verrucomicrobia and reduction in Bacteroidetes in animals that developed HAEC, (HAEC 2 compared with HAEC 0, Figure 5, Tables S2, S3) suggesting that Verrucomicrobia may be contributing to the onset of the HAEC, while Bacteroidetes may protect against enterocolitis. Interestingly, Firmicutes were unchanged (data not shown).

### Genera Taxa Associated with Development of HAEC

**Figure 6:**
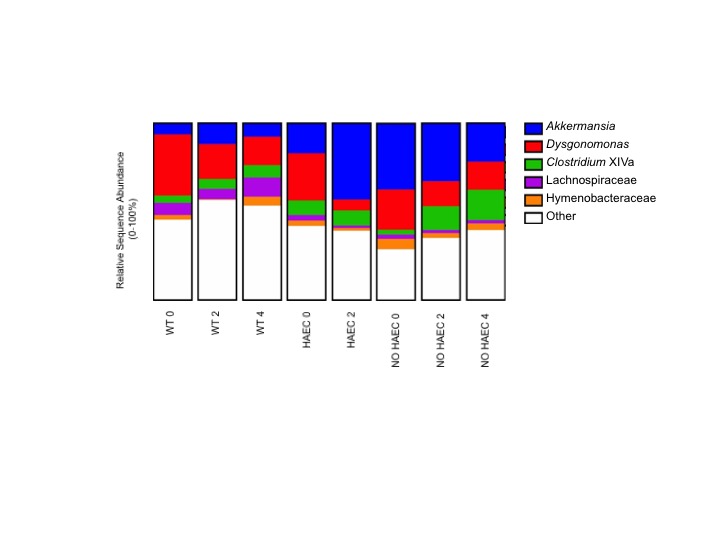
Fecal bacterial genera at several times in relation to pull-through surgery from WT and *Ednrb*^*−/−*^ mice with and without HAEC. Data were generated by analyses of 16S rRNA gene sequences. *n* = 8 (WT 0, 2, 4), 6 (HAEC 0, 2), 4 (NO HAEC 0, 2), 2 (NO HAEC 4).

When we focused on the bacteria at the genera level, we were not surprised to find *Akkermansia* expanded in the animals that developed HAEC (HAEC 2 compared with HAEC 0), given that it falls under the phylum Verrucomicrobia, supporting that *Akkermansia* may be contributing to enterocolitis (Figure 6, Table S4). We further identified lower *Dysgonomonas* in HAEC 2 compared with HAEC 0, suggesting that *Dysgonomonas* may protect against HAEC (Figure 6, Table S5). Interestingly, we also found *Clostridium* XIVa increased in NO HAEC 2 and NO HAEC 4 than NO HAEC 0, suggesting that the process through surgery itself changes the fecal bacterial communities, and that *Clostridium* XIVa may protect against enterocolitis (Figure 6, Table S6).

### Genera Taxa Changes Associated with Pull-Through Surgery

In addition to those genera associated with HAEC, we identified 3 genera associated with pull-through surgery. *Dysgonomonas* was lower in WT 2 and WT 4 than in WT 0, suggesting that the progression through surgery and recovery changes the fecal bacterial communities (Figure 6, Table S5). Conversely, *Hymenobacteraceae* and *Lachnospiraceae* were increased in WT 4 compared with WT 2, further supporting the notion suggesting that the loop through surgery itself alters the fecal bacterial communities (Figure 6 and Tables S7, S8).

## Discussion

A major finding of the study is that *Ednrb^−/−^* mice had distinct bacterial communities from the WT mice indicating that genotype was the primary factor selecting for these differences which was not surprising (17). More striking is the loss of species (OTU) richness associated with development of HAEC and the accompanying shifts in composition of taxa that had not previously been identified. Importantly, the HAEC phenotype had a marked expansion of phyla Verrucomicrobia and its genus *Akkermansia* with reduction of Bacteroidetes compared with the NO HAEC phenotype during the first 2 weeks after pull-through surgery. These novel findings suggests a causal role for *Akkermansia*, a mucin degrading gut commensal found in rodents, humans and many other species (18). While *Akkermansia* has been suggested to have anti-inflammatory properties (19), it has been noted in mouse models to be markedly increased after dextran sulfate sodium (DSS) treatment to induce colitis in multiple studies (20–23) and not in others (24). Recent studies in IBD patients find reduced *Akkermansia *(19, 25). In light of these conflicting reports, it is unclear whether *Akkermansia* was acting as a pathobiotant promoting development of HAEC, or possibly playing a compensatory “protective” role.

Conversely, Bacteroidetes were significantly lower in animals that developed HAEC (HAEC 2). These findings were remarkably similar to a study of the colonic bacteria in children with active HAEC at the time pull-through surgery by Li et al., which also identified reduced Bacteroidetes and a predominance of Proteobacteria compared with children without enterocolitis (6). An earlier study by our group focused on patients with a history of HAEC (but not active HAEC), found the proportion of Bacteroidetes unchanged compared with HSCR patients free from enterocolitis (5). Taken together, these findings suggest that Bacteroidetes may be protective in active HAEC, and may return to baseline for HSCR patients after an HAEC episode. Of note, IBD patients also have reduced Bacteroidetes compared with normal subjects (25) suggesting that lower fecal Bacteroidetes may be associated with gastrointestinal inflammation rather than specific to HAEC. Furthermore, these findings lend support to our surgical mouse model of HAEC robustly mimics HAEC in children.

A new finding in this study was the reduction in the genus *Dysgonomonas* with HAEC (HAEC 2) (Figure 6, Table S5), which has not been previously identified as associated with HAEC in human or animal studies. These findings suggests that *Dysgonomonas* may protect against development of enterocolitis in an acute setting, while a recent study of IBD patients found *Dysgonomonas* to be expanded in Crohn’s disease patients and contracted in those with ulcerative colitis making it unclear of the role it may be playing in chronic gut inflammation (25). Further, we also found *Clostridium* XIVa was elevated in the NO HAEC 2 and NO HAEC 4 groups compared with the NO HAEC 0 group (Figure 6, Table S5) suggesting *Clostridium* XIVa may also be protective against HAEC. Similarly, *Clostridium* XIVa has also been shown to be lower in ulcerative colitis patients compared with normal controls (26).

When we compared our findings with two observational, non-surgical mouse studies utilizing *Ednrb* mutant strains similar to the one used in this study, there was an increased proportion of Bacteroidetes and Proteobacteria, with reduced Firmicutes compared with wild type animals (17, 27, 28). Surprisingly, we found no difference between genotypes in Bacteroidetes or Firmicutes prior to surgery, while there were barely detectible fecal Proteobacteria in our mice (Figure 5, Table S3). Interestingly, as animals in the non-surgical mice developed enterocolitis, the only change was a reduction in proportion of *Lactobacillus* while Bacteroidetes and *Clostridium* remained unchanged (17). We identified lower Bacteroidetes in both the HAEC and NO HAEC groups (Figure 5, Table S3), and increased *Clostridium* XIVa in the NO HAEC groups after surgery compared with WT groups (Figure 6, Table S6), and we did not identify *Lactobacillus* as a significant proportion of the bacterial community in our mice. It is important to point out some major differences in study design likely influenced the intestinal microbial ecology in both of these prior studies compared with our study. A significant difference is that animals in the non-surgical observational studies had untreated colonic obstruction (equivalent to untreated HSCR in humans) contributing to bacterial overgrowth, while in this study, the obstructing aganglionic colon was surgically removed and intestinal continuity reestablished. Another important difference is that in the current study we employed enrofloxacin, a fluoroquinolone antibiotic, around the time of surgery for wound infection prophylaxis which affected the fecal microbial communities. Lastly, the diagnosis of enterocolitis in the prior studies was based solely on subjective clinical observation of animal behavior rather than more objective histological grading of colon tissue for enterocolitis as performed in the current study. This critical difference opens the possibility that “ill-appearing” animals in the prior studies may, or may not, have actually had HAEC. Given these notable differences between study designs, comparing the results of prior non-surgical studies to the current study should be done with caution.

We also identified bacterial communities were altered by the pull-through surgery in the wild type animals (Table S1) and were not associated with enterocolitis. On taxa analysis we found *Dysgonomonas* was lower in WT 2 and WT 4 animals compared with WT 0 (Table S5), while *Hymenobacteraceae* and *Lachnospiraceae* were elevated in WT 4 compared with WT2 (Tables S7, S8). In one study, all three genera were altered after of gastric bypass surgery in adults with type 2 diabetes (29), and elevated *Lachnospiraceae,* a mucus degrading commensal, was recently shown to be associated with anastomotic leak in colorectal operations (30), suggesting complex shifts occur in microbial populations depending on organ system, disease state and type of surgical procedure performed.

Treatment with antibiotics before and after the pull-through operation altered the bacterial microbiome significantly, but was necessary to successfully perform the microsurgical pull-through surgery and achieve post-surgical survival. It is also important to note that treatment with broad spectrum antibiotics is commonly performed in clinical care of children with HSCR prior to pull-through surgery and continued after surgery as well. Hence, our use of antibiotics in this study quite closely mimics clinical care children around the time of pull-through surgery. Nevertheless, these findings leave open the possibility that bacterial microbiome dynamics and changes in diversity play a role in development of HAEC, rather than specific phyla and genera as described DeFilippo et al (31). Alternatively, differences in the fungal and viral microbiomes may provide additional insights into the mechanisms underlying HAEC development but were not evaluated in this study.

Our findings raise the possibility that determination of fecal *Akkermansia* and/or Bacteroidetes proportions in children with Hirschsprung disease prior to their pull-through operation, may be able to identify high-risk patients for developing HAEC after surgery. However, given the obvious difference in species maintained in a controlled environment with SPF conditions and similar chow, which is not representative of the “dirty” world, it is likely that any microbial predictors in humans would be different from the ones identified above. Testing this hypothesis in children would require an appropriately powered and controlled study.

## Conclusion

In summary, we have identified a number of gut bacterial changes associated with the development of HAEC after pull-through surgery. We found increased *Akkermansia* and reduced Bacteroidetes suggesting that these and others may contribute to the HAEC phenotype in this model, and raising the intriguing possibility that there may be pre-surgical microbial markers in humans to identify high risk patients for HAEC. In the future, a prospective human study is needed to explore the microbiome changes that occur in Hirschsprung children around pull-through surgery and with development of HAEC.

## Ethics Statement

The research protocol and all animal care procedures were approved by the Institutional Animal Care and Use Committee (IACUC protocol #2302) at Cedars-Sinai Medical Center.

## Author Contributions

Conceived study and designed the experiments: PF, JB. Performed the experiments: ZC, LZ, DD, PR. Analyzed the data: ZC, PR JB, PF. Contributed reagents/materials/analysis tools: ZC, LZ, DD, PF, PR, JB. Provided funding: PF, JB. Wrote the paper: PF, JB, ZC, RB.

## Conflict of Interest Statement

The authors declare that the research was conducted in the absence of any commercial or financial relationships that could be construed as a potential conflict of interest.

## Supplementary material

Table S1 Adonis Tests of Hellinger Beta Diversity Values from Fecal Bacterial Sequences at Several Times in Relation to Pull-Through Surgery from WT and Ednrb^−^^/^^−^ Mice with and without HAEC.

Table S2 Differences in Relative Sequence Abundance of Fecal Bacteria from the Phylum Verrucomicrobia at Several Times in Relation to Pull-Through Surgery from WT and Ednrb^−/−^ Mice with and without HAEC.

Table S3 Differences in Relative Sequence Abundance of Fecal Bacteria from the Phylum Bacteroidetes at Several Times in Relation to Pull-Through Surgery from WT and Ednrb^−/−^ Mice with and without HAEC.

Table S4 Differences in Relative Sequence Abundance of Fecal Bacteria from the Genus Akkermansia at Several Times in Relation to Pull-Through Surgery from WT and Ednrb^−/−^ Mice with and without HAEC.

Table S5 Differences in Relative Sequence Abundance of Fecal Bacteria from the Genus Dysgonomonas at Several Times in Relation to Pull-Through Surgery from WT and Ednrb^−/−^ Mice with and without HAEC.

Table S6 Differences in Relative Sequence Abundance of Fecal Bacteria from the Genus Clostridium XIVa at Several Times in Relation to Pull-Through Surgery from WT and Ednrb^−/−^ Mice with and without HAEC.

Table S7 Differences in Relative Sequence Abundance of Fecal Bacteria from the Genus Hymenobacteraceae at Several Times in Relation to Pull-Through Surgery from WT and Ednrb^−/−^ Mice with and without HAEC.

Table S8 Differences in Relative Sequence Abundance of Fecal Bacteria from the Genus Lachnospiraceae at Several Times in Relation to Pull-Through Surgery from WT and Ednrb^−/−^ Mice with and without HAEC.

Figure S1 Chao1 rarefaction curves for each group.
