# Supplementary figures and images for: Bacterial Microbiome Dynamics in Post Pull-Through Hirschsprung-Associated Enterocolitis (HAEC): An Experimental Study Employing the Endothelin Receptor B-Null Mouse Model
Source: Front Surg. 2018 Apr 6;5:30. doi: 10.3389/fsurg.2018.00030 (PMC5897423; doi:10.3389/fsurg.2018.00030)

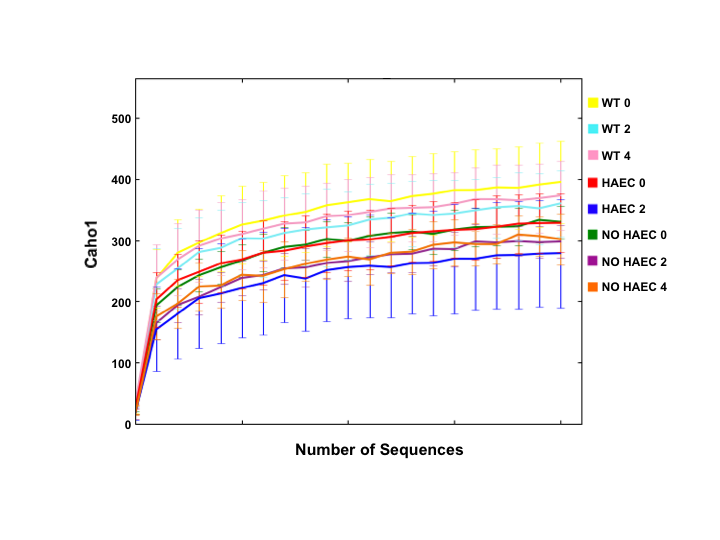

Supplement: Figure S1 — Chao1 rarefaction curves for each group. [file Image1.TIFF]
